# Bilateral Ovarian Torsion Due to a Giant Mucinous Cystadenoma, Contralateral Mature Teratoma, and Paratubal Cyst in a Young Adult

**DOI:** 10.7759/cureus.44913

**Published:** 2023-09-08

**Authors:** Lucas J Betts, Kalley Johnson, Emma Bassette, Cameron Slife, Jimmy Khandalavala

**Affiliations:** 1 Radiology, Creighton University School of Medicine, Omaha, USA; 2 Plastic Surgery, Creighton University School of Medicine, Omaha, USA; 3 Obstetrics and Gynecology, Creighton University School of Medicine, Omaha, USA

**Keywords:** controlled fluid aspiration, mature teratoma, ovarian mass, mucinous cystadenoma, bilateral ovarian torsion

## Abstract

Giant ovarian cysts (>10 cm) are rare due to the widespread use of routine imaging. However, in the absence of compressive symptoms, giant cysts remain a diagnostic challenge, especially in patients with larger body habitus. Complications of benign ovarian cysts are infrequent but can include torsion of the adnexa. Bilateral torsion is a rare emergency that can threaten a patient’s future fertility. In this case, we report on a 24-year-old female with bilateral torsion due to a triad of adnexal masses including a 30 cm mucinous cystadenoma, a 10 cm mature teratoma, and an 8 cm paratubal cyst. Controlled fluid aspiration was performed prior to en bloc resection of the cystadenoma due to the emergent nature of the case and lack of malignant features.

## Introduction

Giant ovarian cysts (>10 cm) are rare given the widespread use of routine imaging [1]. Benign ovarian cysts are often asymptomatic and do not present until compression of surrounding structures occurs, leading to a late diagnosis. If symptomatic, the most common presenting symptoms include fatigue, bloating, increased abdominal size, abdominal/pelvic pain, urinary symptoms, and constipation [2]. Additionally, abdominal distension due to cyst growth can remain undetected to the patient and clinician in the setting of obesity.

Of the benign subtypes of ovarian masses, approximately 60-67% are serous cystadenomas, 19-20% are mucinous cystadenomas, and 11-14% are mature cystic teratomas [3,4]. Paratubal cysts account for 10% of all adnexal masses, with an estimated incidence of 3% of the general population [5,6]. The most common complications of benign ovarian cysts are torsion, rupture, and hemorrhage, although these are rare. Bilateral torsion is very rare but can have catastrophic consequences regarding future fertility [7]. In this case, we report on a 24-year-old with bilateral ovarian torsion secondary to concurrent giant mucinous cystadenoma, contralateral teratoma, and paratubal cyst.

## Case presentation

A 24-year-old, obese (BMI = 35 kg/m^2^), nulligravid female with no significant past medical history presented to an outside emergency department (ED) with nonspecific, intermittent abdominal pain. The pain had been gradually increasing over the course of six months but fluctuated in severity. Urinalysis, complete metabolic panel, and complete blood count were all within normal limits. The patient reported normal menses, no systemic symptoms, and normal bowel and bladder function. The pain was relieved with ketorolac and presumed to be musculoskeletal in nature. Primary care follow-up a week after the ED visit also revealed no abnormalities.

**Figure 1:**
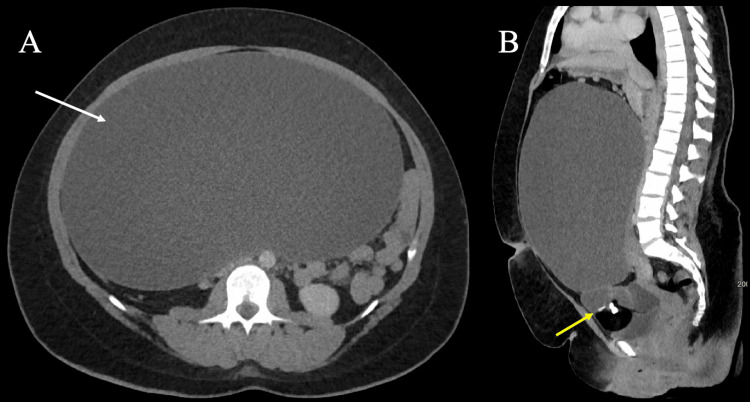
(A) Axial and sagittal CT images of the abdomen/pelvis revealed a 30 x 17 x 30 cm cystic mass (white arrow) spanning the abdomen and displacing the adjacent viscera. (B) The mass appeared to have loculated areas of calcification and fat at the inferior pole (yellow arrow) and large swirling vessels.

One month later, the patient returned to the ED with acute worsening of her abdominal pain, especially on the left side. The pain was unrelieved by ketorolac and morphine in the ED. CT scan of the abdomen and pelvis revealed a large cystic mass appearing to arise from the left ovary, measuring 30 x 17 x 30 cm with loculated areas of calcification, fat density, and large swirling vessels. CT also revealed another 8 x 5 cm cystic mass of unknown origin located inferior to the larger cyst (Figure 1).

Left ovarian torsion was suspected, and emergency open exploratory laparotomy was performed via a midline incision. The large 30 cm left ovarian cyst was encountered first and was obstructing visualization of the right adnexa. Due to the emergent nature of suspected ovarian torsion, the large size of the cyst, and the lack of malignant features (i.e. solid, thick-walled, poorly defined margins, ascites), the decision was made to proceed with controlled fluid aspiration of the cyst prior to left salpingo-oophorectomy. Eight point five (8.5) liters of straw-colored clear fluid was removed from the left-sided cyst and torsion of the left adnexa was confirmed.

**Figure 2:**
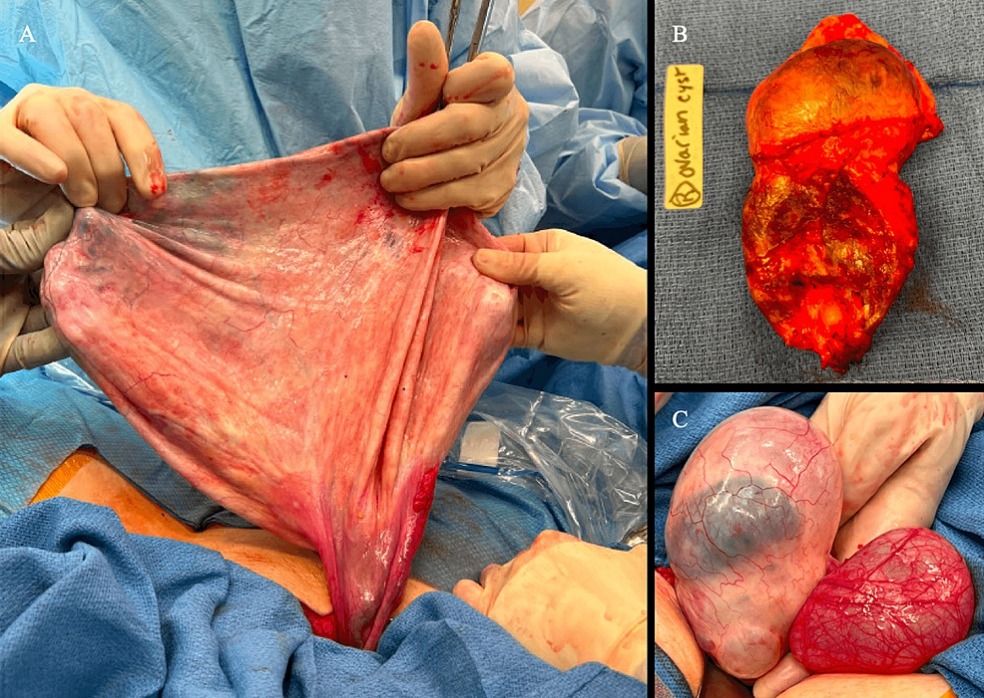
Intraoperative images from the exploratory laparotomy (A) Giant mucinous cystadenoma of the left ovary post controlled aspiration of 8.5 L of clear, straw-colored fluid. (B) Mature cystic teratoma of the right ovary. (C) Paratubal cyst of the distal right fallopian tube.

Upon further exploration of the pelvis, the right adnexa was also found to be torsed. The right adnexa contained an 8 cm paratubal cyst and a 10 cm ovarian cyst with hair, teeth, and caseous fluid. Left salpingo-oophorectomy, right paratubal cyst excision, and right ovarian cystectomy were performed with right ovarian preservation. Histologic diagnosis revealed a left ovarian mucinous cystadenoma, a right paratubal cyst, and a right ovarian mature teratoma (Figure 2). A follow-up visit a week after the surgery revealed no postoperative complications.

## Discussion

Giant ovarian cysts pose a diagnostic challenge due to their rarity, nonspecific presentation, and broad differential diagnosis. The differential of a giant intra-abdominal cyst includes pregnancy, ascites, or cysts originating from other abdominal organs such as the liver, pancreas, omentum, or biliary tree.

Differentiation of benign versus malignant ovarian masses is critical for optimal clinical management. If malignancy is suspected, patients should receive timely referral to gynecologic oncology due to the survival benefits of cytoreductive surgery and chemotherapy [8-10]. Additionally, the use of a risk of malignancy index tool may help optimize surgical planning and the treatment approach [11]. However, in the setting of suspected acute adnexal torsion, our patient was not able to benefit from a malignancy risk assessment with further imaging and CA-125 measurement prior to undergoing exploratory laparotomy.

In the setting of giant ovarian cysts, the most commonly reported treatment is en bloc resection with or without controlled aspiration of cyst contents. Some authors advise against pre- or intraoperative controlled aspiration due to the increased risk of bleeding, infection, and spillage of potentially malignant cells [12,13]. However, one major benefit to controlled aspiration prior to en bloc resection is the decreased risk of supine hypotension syndrome and splanchnic shock due to rapid hemodynamic shifts [14]. Due to the emergent nature of our case, the giant size of the cyst, and the lack of malignant features on CT, the decision was made to proceed with controlled aspiration prior to en bloc resection.

Frozen sections and histopathology are used to confirm the diagnosis of ovarian masses [15]. The exact histogenesis of mucinous cystadenomas remains unclear, but the leading proposed theories suggest a surface epithelial metaplasia origin versus a teratomatous origin [16]. Mucinous cystadenomas are associated with a concurrent mature cystic teratoma in approximately 10% of cases, which serves as the basis of the teratomatous origin theory [17]. There do not appear to be genetic associations of either tumor with paratubal cysts.

Giant mucinous cystadenomas have rarely been reported, especially in the setting of concurrent adnexal tumors [18-20]. In our patient, the pathologic diagnosis revealed a triad of large adnexal masses, including a 30 cm mucinous cystadenoma, a 10 cm mature teratoma, and an 8 cm paratubal cyst. The presence of multiple large undetected cysts resulted in bilateral torsion in our patient, another rarely reported phenomenon that immediately threatens future fertility.

## Conclusions

Giant ovarian cysts are rarely seen due to the widespread availability and use of imaging. Given their asymptomatic nature, giant ovarian cysts often present late due to compressive symptoms and can remain undetected in the setting of obesity. Giant mucinous cystadenomas have rarely been reported and can cause adnexal torsion despite their size. Clinicians need to maintain diagnostic awareness about giant ovarian cysts. Optimal management includes timely referral to a gynecologic oncologist if malignancy is suspected and a risk of malignancy index assessment prior to treatment if not in an emergency setting. A giant ovarian cyst in the setting of adnexal torsion and no malignant features can be managed safely with controlled aspiration prior to en bloc resection.
